# Analyzing fast and slow: Combining traditional and rapid qualitative analysis to meet multiple objectives of a complex transnational study

**DOI:** 10.3389/fsoc.2023.961202

**Published:** 2023-02-01

**Authors:** Lauren Suchman, Elizabeth Omoluabi, Julia Kramer, Janelli Vallin, Erica Sedlander, Serah Gitome, Pauline Wekesa, Zachary Kwena, Rachel Granovsky, Agnes Kayego, Betty Kaudha, Lynn Atuyambe, Dinah Amongin, Phoebe Alitubeera, Aminat Tijani, Chioma Okoli, Ayobambo Jegede, Martha Kamanga, Mandayachepa Nyando, Louisa Ndunyu, Kelsey Holt, The ICAN Research Consortium, Kelsey Holt

**Affiliations:** ^1^University of California, San Francisco, San Francisco, CA, United States; ^2^AkenaPlus Health, Abuja, Nigeria; ^3^Kenya Medical Research Institute, Nairobi, Kenya; ^4^Makerere University School of Public Health, Kampala, Uganda; ^5^Malawi University of Science and Technology, Limbe, Malawi; ^6^Maseno University, Kisumu, Kenya

**Keywords:** rapid analysis, qualitative research, human-centered design, global health, Kenya, Uganda, Malawi, Nigeria

## Abstract

Much of the methodological literature on rapid qualitative analysis describes processes used by a relatively small number of researchers focusing on one study site and using rapid analysis to replace a traditional analytical approach. In this paper, we describe the experiences of a transnational research consortium integrating both rapid and traditional qualitative analysis approaches to develop social theory while also informing program design. Research was conducted by the Innovations for Choice and Autonomy (ICAN) consortium, which seeks to understand how self-injection of the contraceptive subcutaneous depot medroxyprogesterone acetate (DMPA-SC) can be implemented in a way that best meets women's needs, as defined by women themselves. Consortium members are based in Kenya, Uganda, Malawi, Nigeria, and the United States. Data for the ICAN study was collected in all four countries in sub-Saharan Africa. In order to both illuminate social phenomena across study sites and inform the program design component of the study, researchers developed tools meant to gather both in-depth information about women's contraceptive decision-making and data targeted specifically to program design during the formative qualitative phase of the study. Using these two bodies of data, researchers then simultaneously conducted both a traditional qualitative and rapid analysis to meet multiple study objectives. To complete the traditional analysis, researchers coded interview transcripts and kept analytical memos, while also drawing on data collected by tools developed for the rapid analysis. Rapid analysis consisted of simultaneously collecting data and reviewing notes developed specifically for this analysis. We conclude that integrating traditional and rapid qualitative analysis enabled us to meet the needs of a complex transnational study with the added benefit of grounding our program design work in more robust primary data than normally is available for studies using a human-centered design approach to intervention development. However, the realities of conducting a multi-faceted study across multiple countries and contexts made truly “rapid” analysis challenging.

## 1. Introduction

The methodological literature on rapid qualitative analysis generally focuses on studies that have: (1) solely employed rapid techniques; (2) used both rapid and traditional approaches for the purpose of comparing the two in terms of their ability to generate reliable findings (Taylor et al., 2018; Nevedal et al., 2021). Combining these two analytical approaches and using them complementarily can be useful for projects that have a program design element in addition to a desire to illuminate social phenomena. However, despite the promise of leveraging qualitative data for myriad project objectives, we know little about what it looks like to integrate rapid and traditional analytical approaches, and how this integration can serve researchers working on various aspects of a single study.

While there is a range of methods qualitative researchers can draw on to analyze their data (Huberman and Miles, 1994), these methods generally entail organizing data *via* coding, and then iteratively developing a set of themes through several stages of data review and comparison (Lester et al., 2020). The amount of time this process can take varies widely depending on the method chosen, the amount of data to be analyzed, and the number of researchers participating in the analysis. Particularly because traditional qualitative analysis can be very time- and resource-intensive (Queirós et al., 2017), and often relies on a small group of researchers' deep knowledge of the data, researchers leading complex global health studies need versatile solutions that will enable multiple researchers on a team to use qualitative findings to inform multiple study components.

Rapid qualitative analysis is one potential solution, as these methods can help to: reduce study time and cost; improve data collection efficiency and accuracy of findings; and collect a large amount of data in a reduced period of time (Vindrola-Padros and Johnson, 2020). Since producing written transcripts is one of the most time-consuming aspects of qualitative analysis, as Vindrola-Padros and Johnson (2020) note, most researchers employing rapid techniques focus on either eliminating the use of transcripts through techniques such as mind-mapping or coding directly from audio/visual sources (Halcomb and Davidson, 2006; Burgess-Allen and Owen-Smith, 2010; Neal et al., 2015), or turning out transcripts more quickly using either specialized equipment or specialists who are trained in fast transcription (Scott et al., 2009; Johnson, 2011). Studies analyzing the validity of these approaches have found that results tend to be equally valid regardless of approach (Gale et al., 2019; Nevedal et al., 2021) as long as researchers are skilled in using relevant techniques and software (Davis and Meyer, 2009). However, while there is an established evidence base surrounding rapid analysis, qualitative researchers are underutilizing the potential to combine these approaches with more traditional qualitative work to speak to social phenomena while addressing applied study objectives at the same time.

With its promise to quickly design programs that are uniquely responsive to users' needs, human-centered design (HCD) is a burgeoning area in the global health field. HCD researchers and practitioners seek to iterate and “fail fast to succeed sooner” in their design and iteration of solutions (Thoring and Müller, 2011; Müller and Thoring, 2012; Brown, 2013). Altman et al. (2018) note that this philosophy of rapid failure can pose a tension in the health field, where the stakes of failure are high. However, while Altman et al. note this tension between design and health, they also note the role that *low-stakes* failure (e.g., low-fidelity prototyping, testing, and analysis early in the design process) can minimize the risks of end-of-process failure. Therefore, rapid analysis of qualitative data is a key component of the HCD methodology, as it plays a role in moving quickly between information gathering and solution development/testing. However, strategies for rapid analysis in the design process are rarely discussed in the literature (Roschuni et al., 2015). HCD researchers instead write about “sensemaking” as an activity that happens intuitively through iterative reflection on the part of both individuals and research teams (Kolko, 2010a,b; Stigliani and Ravasi, 2012). Despite both qualitative and HCD research employing rapid analysis methods for similar ends, to our knowledge the literature on rapid qualitative analysis has had little overlap with the literature on HCD. This is an area of opportunity to better understand how rapid qualitative analysis might best be integrated into HCD work as part of a multi-faceted study.

Below, we describe the experiences of a multi-country consortium combining both rapid and traditional qualitative analytical approaches to: (1) investigate women's experiences making decisions regarding contraception in Kenya, Uganda, Malawi and Nigeria; (2) use an HCD approach to develop interventions that will facilitate women's access to a new self-injectable contraceptive in Kenya, Uganda and Malawi. We detail the processes we used to conduct both traditional qualitative analysis across multiple teams, sites and contexts, and the ways in which findings from both this analysis and a rapid analysis process contributed to the program design component of the overall study.

## 2. The ICAN study

Launched in late 2019, the Innovations for Choice and Autonomy (ICAN) study aims to understand how self-injection of the contraceptive subcutaneous depot medroxyprogesterone acetate (DMPA-SC) can be implemented in a way that best meets women's needs, as defined by women themselves. ICAN research consortium partners are based at: the Malawi University of Science and Technology (MUST) in Malawi; the Makerere University School of Public Health (MakSPH) in Uganda; the Kenya Medical Research Institute (KEMRI) and Maseno University in Kenya; AkenaPlus Health in Nigeria; and the University of California San Francisco (UCSF) in the United States.

Each ICAN site has formed its own core team that consists of 1–2 lead researchers working closely with at least two senior researchers, and varying numbers of junior researchers and a flexible number of support staff depending on the scope of work to be carried out locally. In total, there are 8–12 members of each core team in the consortium. Project leads in each ICAN country selected team members with a mix of expertise in qualitative and quantitative methods, program intervention design, and project management to meet the diverse needs of the study. The US-based team, as prime funding recipient, is responsible for cross-country research design, and overall project oversight and management. Each team based in sub-Saharan Africa does the same at country level while managing local stakeholder engagement, data collection, and ethical board approvals. These teams have the agency to adapt and localize agreed upon methodology and timing of data collection based on their own deep understanding of the local environment. All teams are responsible for contributing to overall consortium governance, research design, data collection, data analysis, publication, and support for early career researchers.

To achieve the overall study objective, the ICAN study was divided into two phases with distinct aims. The aim of Phase 1 is to deeply understand contraceptive decision-making and women's experiences seeking, accessing, and using contraception, to understand for whom self-injection of DMPA-SC may be a powerful method. The aim of Phase 2 is to identify effective approaches for introducing and supporting the use of self-injection (in the context of a variety of contraceptive options) in a way that helps women overcome barriers and optimize facilitators to contraceptive decision-making and use.

### 2.1. Phase 1: Formative qualitative research

This paper focuses on Phase 1 of the ICAN project as well as the early stage of Phase 2. The Phase 1 formative qualitative research involved first collecting 241 (approximately 60 per country) semi-structured in-depth interviews with women of reproductive age in the ICAN countries located in sub-Saharan Africa: Kenya; Uganda; Malawi; and Nigeria. Women were purposively sampled based on their age, prior contraceptive use or non-use, and previous experience with DMPA-SC. In the age category, our sample was divided between age groups 15–19 years and ages 20–45 years because adolescents often have different attitudes toward and experiences with contraception than older, often married, women. In each country, data collection took place in two geographically and culturally diverse sites (Nairobi and Kisumu metropolitan areas in Kenya; Oyam and Mayuge districts in Uganda; Ntchisi and Mulanje districts in Malawi; and Enugu and Plateau states in Nigeria). Teams used various methods to recruit participants in each site, including working with either local community health volunteers/workers, local health providers, or members of the local ICAN Community Advisory Boards to identify potential participants. Data collection was conducted by ICAN team members who were fluent in the local language and trained in qualitative research. The data collection instruments included questions meant to help the researchers: (1) understand women's contraceptive decision-making and opinions related to self-injection of DMPA-SC (Phase 1 research priorities); (2) identify effective approaches for introducing and supporting the use of self-injection (Phase 2). All participants provided either verbal or written consent to be interviewed depending on local ethical requirements and interviews took an average of 1 h to complete. The analysis phase of the research is described in detail below.

### 2.2. Phase 2: Program design

In Phase 2 of the ICAN study, we carried out a human-centered design process in three of the ICAN study countries: Kenya, Uganda, and Malawi. In each country, the ICAN team aimed to identify effective approaches for introducing and supporting the use of self-injection in a different sector: the e-commerce sector in Kenya, women's social communication networks in Uganda, and health surveillance assistants in Malawi. These sectors were chosen strategically based on where ICAN would best be able to support other self-injection work led by grantees of the same funder. We structured our human-centered design approach around the following stages: (1) conduct design research, (2) analyze design research, (3) generate ideas, (4) create testable prototypes, (5) test prototypes with stakeholders, and (6) refine and finalize prototypes for implementation.

Stages 1 and 2 of this HCD process sought to complement the broad research conducted in Phase 1 of the ICAN study to include a specific focus on the channel of interest in each country. Therefore, the rapid analysis process described in this paper was leveraged to help inform the human-centered design process undertaken in Phase 2 of the ICAN study.

## 3. Integrating traditional and rapid qualitative analysis to meet multiple study goals

While ICAN has followed a traditional qualitative analysis approach to answer key research questions related to the ways in which women make and act on decisions related to contraceptive use, the team also has strategically employed rapid analysis techniques to respond to the many needs of a complex study. Below, we detail the processes we used to meet multiple study objectives using one set of in-depth interviews.

### 3.1. Simultaneous data collection and preliminary analysis

To meet the first objective of the ICAN study—deeply understanding contraceptive decision-making and women's experiences seeking, accessing, and using contraception—we employed a traditional approach to qualitative data analysis. In order to make this process work across a complex research consortium, some of the larger ICAN teams appointed a subset of researchers (about 4–5 people) to a local qualitative analysis team, while some teams elected to have all of their members participate in the analysis team. Members of ICAN US also joined each local team. In each case, teams consisted of researchers who specialize in qualitative methods as well as researchers who are interested in growing their skillset in this area. We describe the process of organizing a traditional analysis across multiple countries, sites and teams in more detail in a separate manuscript that is currently in preparation (Suchman et al., in preparation). Manuscripts describing our findings are in preparation as well.

Since one of our goals in meeting our first study objective was to develop a theory of women's contraceptive decision-making, we adopted a modified Grounded Theory approach. Grounded Theory involves constant engagement with analysis throughout the data collection process and beyond, requiring researchers to iteratively collect, analyze, and question data until they have reached saturation for the key themes that inductively arise (Glaser and Strauss, 1967; Corbin and Strauss, 1990; Charmaz, 2006). Though Grounded Theory often requires multiple rounds of data collection to allow for iterative theory development, fully adopting this approach was not practical for our multi-country team due to time constraints and IRB constraints regarding modifications to study instruments.

We therefore modified the Grounded Theory approach by dividing qualitative data collection into four stages in Kenya, Uganda and Malawi. This staged approach included pauses between each stage to allow the qualitative teams to begin tracking emerging themes in the data, monitor data quality, and make adjustments to data collection instruments, as appropriate and allowable by local IRBs, while fieldwork was being conducted. While all three ICAN country teams followed the same set of phases, the timing of data collection was staggered across countries with ICAN Kenya collecting data from February–April 2021, ICAN Uganda collecting data from February–May 2021, and ICAN Malawi collecting data from March–June 2021. Further demonstrating the potential limitations of Grounded Theory methodology in some settings, the ICAN Nigeria team did not consider a lengthy data collection period to be either practical or safe, and after rigorous planning and piloting, conducted data collection over a 2-week period in September 2021. Since the data collection instruments had been tested extensively by other ICAN teams at that point (and were further tested and refined by the ICAN Nigeria team prior to launching data collection), the team scheduled data collection within the shortest time practicable to assure the safety of field staff while maintaining the integrity of the data.

Our approach to the pauses between each data collection stage combined the Grounded Theory practice of “open coding” with an approach adapted from the literature on rapid qualitative analysis to improve efficiency of data review. In contrast to more structured qualitative analysis approaches, which entail developing a key set of themes to explore in the data before coding (Ritchie et al., 2014), open coding involves reading through full interview transcripts and noting key themes as they emerge from the data. As themes emerge, they may necessitate additional data collection to ensure that researchers have adequate data to support the validity of each theme (Walker and Myrick, 2006). Once ICAN data collectors completed an audio-recorded interview, it was immediately sent for transcription. As completed transcripts became available, they were shared with the larger team in a secure, cloud-based folder (using Box content management software) and members of each qualitative analysis team were assigned one or two full transcripts to read and open code during each data collection phase. This process started slowly in Phase One of data collection, because most transcripts in each country had to be simultaneously transcribed and translated into English so that team members across all ICAN countries could use the full dataset.

On the rapid analysis side, interviewers completed a post-interview report form (PIRF) following each interview (see Supplementary material for the PIRF template). Depending on what local teams determined made the most sense for their team in the field, some teams used paper-based versions of the PIRF form, while others completed the form in the survey instrument REDCap. REDCap was accessible on interviewers' laptops or mobile phones either on- or offline, and this ability to complete the PIRFs without internet access was key for interviewers working in remote areas. Once an interviewer connected to the internet, data was immediately transferred to the shared REDCap server. One ICAN team member monitored REDCap for all countries and regularly transferred completed PIRFs to a shared Box folder so that all team members could access them. Paper-based PIRFs were returned to each team's office and scanned into a shared folder.

The PIRFs were designed using a combination of the framework approach that some qualitative researchers have used to quickly analyze data that has already been transcribed (Fox et al., 2016; Koenig et al., 2016; Palinkas et al., 2019), as well as the field notes qualitative researchers often use to contextualize their analysis (Phillippi and Lauderdale, 2018). These structured note-taking forms covered seven key questions related to ICAN's main areas of inquiry. In line with our first objective to deeply understand and theorize women's contraceptive decision-making, the PIRFs were meant to help us move data analysis along more efficiently. We found that they helped our teams avoid a heavy dependence on completed transcripts to conduct our modified Grounded Theory approach during the phased data collection process, which was especially important given the often slow pace of simultaneous transcription and translation. In line with our second objective, the PIRFs also allowed us to quickly analyze a subset of data to contribute to the HCD process, which began in Kenya and Uganda shortly after we completed data collection.

During each pause, analysis team members skimmed the PIRFs collected since the last pause in addition to reviewing and open coding their assigned full transcripts. Analysis teams then met and used a structured meeting guide (see Supplementary material) to facilitate discussion of findings as related to the study's key research questions. Teams also discussed additional themes emerging from the data through the open coding process and any suggested adjustments to the data collection instruments to better capture emergent themes and ensure accuracy of the data. Data collection instruments were then updated as needed and allowed by local IRBs before fieldwork resumed.

### 3.2. Modifying a traditional approach to coding and writing analytic memos

Once data collection was complete in each country, we took the traditional approach of coding our qualitative data (Basit, 2003; Williams and Moser, 2019; Giesen and Roeser, 2020). During this phase, each of the coding teams based in Kenya, Uganda, Malawi and Nigeria worked to establish consistent code application using a codebook that was co-developed both inductively drawing on emergent themes from the data collection pauses, and deductively using the available literature. Using a process developed by the ICAN Kenya team, members of each analysis team used Dedoose qualitative analysis software to individually code the same transcript and then met as a group to code the same transcript together over a series of Zoom sessions. ICAN US team members joined these meetings to facilitate cross-country learning and sharing. After coding a full transcript as a group, individual team members then coded a second transcript and split into pairs to discuss any questions or discrepancies in coding. All teams achieved consistent code application after these two rounds and then moved on to coding independently.

To complete independent coding, a subset of researchers from the original analysis teams used Dedoose to code a set of individually assigned transcripts from their own country.

The ICAN US team developed a standardized template for analytic memos that was meant to help coders capture preliminary findings related to key study themes in real time while coding. These memos were divided by key research questions, such as “How do women form contraceptive preferences?,” each of which had related sub-questions. However, coders found these lengthy templates cumbersome and impractical, and they were rarely used. Instead, some researchers kept more flexible memos of their own. Unlike the memos used in Grounded Theory (Montgomery and Bailey, 2007), these memos largely consisted of bullet points related to both key study themes and any emerging themes with supporting quotes, rather than reflecting more deeply on the research process and findings. This bulleting process was less time-consuming than reflective memoing and acted as a way to summarize takeaways from individual interviews with some synthesis. Memos were saved in a shared Box folder so that all team members are able to access them, and several ICAN researchers have already used these notes to inform their own analyses without having to refer back to many code reports.

Final analyses are ongoing and are driven by key ICAN research questions, as well as the individual interests of ICAN team members. Each ICAN team has decided which set of analyses they would like to prioritize and individual researchers across countries are leading both country-specific and cross-country analyses.

### 3.3. Rapid analysis for program design

While the qualitative analysis teams were working toward consistent code application and beginning independent coding in late 2021, researchers participating in the ICAN HCD workstream in Kenya, Uganda and Malawi were beginning the program intervention design phase (Phase 2) of the project. In Nigeria, ICAN is conducting implementation research and evaluation of two existing programs that aim to support providers offering DMPA-SC rather than developing a new intervention.

Before launching program design, a team of ICAN researchers from each country conducted a rapid desk review of the literature relevant to the service delivery channel they expected to focus on. In Kenya, ICAN has partnered with the online pharmacy Kasha (www.kasha.co.ke) to bolster the dissemination of DMPA-SC in the e-commerce space, while the project is partnering with Malawi's Ministry of Health to support community health workers in offering DMPA-SC for self-injection. In Uganda, ICAN has partnered with the AIDS Information Center (AIC) and the Baitambogwe Community Healthcare Initiative (BACHI) to support women in making and acting on contraceptive decisions by leveraging social communication networks outside the healthcare system. The first step in the desk review process was to brainstorm topics and questions to better define the design challenge in each country. After agreeing on a list of relevant topics, representatives from each team were assigned a topic to research. They then sought out papers relevant to their assigned topics using internet searches and by searching through the team's existing database in the Zotero citation management program, which was accessible to all members. After finding relevant papers, team members completed a quick summary of the article in a shared Google sheet. Google sheets was used to account for version control and to ease collaboration. During this process, journal articles were shared amongst team members using a shared Box drive and also were saved in Zotero. The literature review was bolstered by review of the PIRFs completed during qualitative data collection in Phase 1, as well as review of a subset of qualitative transcripts. These materials also were divided up among HCD team members, who reviewed them individually and completed a shared Word document summarizing their findings. Based on the set of information gathered, the desk review for each country was summarized into a word document utilizing a socioecological framework. HCD teams used this data to identify key themes and data points that helped to frame and refine the HCD research question, and also to inform subsequent steps in the design process.

Building on the desk review, several members of the research team who had been more actively involved in earlier phases of qualitative analysis conducted a rapid review of an additional subset of full transcripts as well as an additional set of PIRFs from each country, and developed insights to complement the desk review findings and directly inform intervention design. While the desk review focused narrowly on each country's channel of interest to gain a specific understanding of how women engage with the various channels, this additional rapid review used a broader lens to incorporate data related to women's contraceptive decision-making (collected mainly to answer our Phase 1 research question) with the goal of providing a more holistic understanding of the context in which women will potentially interact with and use the chosen delivery channel in each country. The three researchers selected the PIRFs and transcripts for review with the goal of equally representing contraceptive users and non-users, with some over-sampling of DMPA-SC users. They then divided and assigned the PIRFs and transcripts equally among themselves, and each developed analytic memos similar to those used for the full qualitative analysis with a focus on key themes that were relevant to intervention design. The researchers met regularly during this process to discuss and consolidate findings before sharing a final draft with the individual HCD teams *via* email and the shared Box drive. Members of the HCD teams then discussed and provided feedback on the preliminary insights before they were formally adopted into the intervention design process.

This rapid understanding was critical at multiple points in the HCD process: first, the rapid analysis directly informed the plan to collect additional data (stage 1 of HCD), and second, the rapid analysis was directly relevant to developing and prototyping new solutions (stages 3 and 4 of HCD).

First, the rapid analysis informed additional data collection in the HCD process by quickly summarizing key themes from interviews related to each country's channel of interest (e-commerce in Kenya, community health workers in Malawi, and social communication networks in Uganda). These themes were then used to identify areas where HCD data collection needed to go further to better understand the sector-specific constraints and opportunities in each country. For example, in Kenya, a rapid analysis of the initial in-depth interviews showed a theme around respondents being open to shopping online, but also not fully trusting the online shopping experience. The articulation of this theme led the ICAN HCD team in Kenya to probe deeper into the balance between motivators and barriers to shopping online: how big of a challenge is lack of trust in e-commerce, and for whom does the benefits of shopping online outweigh the barriers to doing so?

Second, the rapid analysis informed solution development in the HCD process by helping to examine the most appropriate or effective message around contraception, messenger to deliver the message, and mode of communication for the intervention. For example, preliminary findings from the ICAN qualitative data suggested that women in Uganda trusted healthcare providers above other sources, such as friends and media, to give them reliable health information. Since the ICAN project will be developing an intervention that uses social networks to provide peer support and convey information related to contraception in Uganda, the qualitative team recommended that any intervention using social networks still employ healthcare providers in some way to convey information critical to the program's success.

Following this analysis process, stages 3 through 6 of the human-centered design process sought to create new and novel solutions to address the needs identified from our research. While we do not describe our process of solution generation in this article, we note that a core value of the HCD process is in leveraging a deep understanding of users' needs and contexts to then drive a creative process of developing a wide range of solutions and then testing and iterating to find solutions that are feasible, viable, and desirable (Brown and Katz, 2009).

## 4. Discussion

Through a combination of rapid and traditional qualitative analysis techniques, we were able to meet multiple objectives of a complex study and ground our human-centered design process in more robust data than is normally available for this type of intervention design work. In Phase 1 of the ICAN study, we adapted a modified grounded theory approach in three of the four ICAN study countries. The outputs from this phase were translated and transcribed in-depth interviews, a subset of transcripts that were open coded by research team members, the post-interview report forms (PIRFs) that summarized key points of each interview, and notes from qualitative analysis team meetings conducted during each data collection pause. These outputs were used during data collection to inform needed adjustments to the interview guides and all transcripts are currently under additional analysis to answer key study questions related to women's contraceptive decision-making. In addition, the transcripts and PIRFs collected during Phase 1 of the study were used in a rapid analysis process to inform the initial stages of the intervention design work conducted in Phase 2. Since human-centered design typically employs only a rapid approach to both data collection and analysis (IDEO.org, 2015), drawing on a large qualitative sample to contextualize the program design work allowed for more robust findings to feed into the HCD workstream. This gave the team greater confidence in the solutions they were developing and testing, and sets the stage for designing interventions that are more attuned to user needs and less likely to require extensive adaptation.

While using rapid analysis techniques helped our team stick to timelines and share data more efficiently across a large group of researchers, our experiences also highlight the limits of rapid analysis in a multi-faceted project carried out across a variety of settings. First, because much of the methodological literature on rapid analysis suggests bypassing the transcription process through means such as listening directly to interview audio recordings or using specialized transcription software, it presumes that interviews are conducted in a language that is: (1) familiar to the researchers; (2) legible to voice recognition software. These conditions are often not met in global health studies, particularly when working in transnational teams. In the ICAN study, all interviews were conducted in a local language and then had to be both translated and transcribed to make them accessible to researchers working across all five ICAN countries. In some cases, such as in Nigeria, even ICAN researchers based in Abuja did not speak the local languages in the chosen study settings, and had to hire and train interviewers with these linguistic skills to conduct data collection. Given the amount of time it takes to translate, transcribe and quality check interviews that often lasted 60 mins or more, as well as the amount of data under review (~60 interviews per country), a rapid review of all transcripts would not have been possible during Phase 1 of the study. Although we did not fully anticipate this challenge when we developed the PIRFs, these forms ended up being critical for quickly gathering usable data. We were then able to triangulate this data with findings from open coding of a small subset of available transcripts to develop preliminary themes and inform additional data collection.

Further, many of the studies described in the rapid analysis literature rely on relatively small, local teams. In HCD work, this is a requirement to ensure that resulting programs are tailored to local context (Melles et al., 2021). However, a significant proportion of global health studies employ researchers working across international borders. Some rapid qualitative research conducted using large teams spread across multiple locales suggests that it can be challenging due to the increased administrative burden of managing multiple teams at once (Vindrola-Padros et al., 2020), which can diminish the time savings that rapid approaches are meant to offer. We concur that managing qualitative analysis across multiple teams created a significant burden for the coordinating team at ICAN US. In addition, because researchers from ICAN US had to join many of the individual analysis team meetings for the purposes of coordination, this likely slowed down an analysis process that might have been more efficient if managed locally or in a way that allowed all analysis teams to coordinate more organically amongst themselves. To this extent, Vindrola-Padros et al. (2020) decision to allow individual teams a significant amount of autonomy may be an attractive alternative for the sake of efficiency. In addition to being efficient, it is critical that all teams have the autonomy required to respond to local conditions in a way that keeps researchers safe. This was demonstrated in our own study by the ICAN Nigeria team's decision to conduct data collection in a relatively short timeframe due to security reasons. This was also the case for numerous studies conducted in the context of the COVID-19 pandemic (Omary et al., 2020). However, we note that while autonomy is critical, cross-country analysis is an opportunity to bring teams back together to collaborate and develop analyses that are greater than the sum of their parts.

The rapid analysis literature also suggests that conducting rapid analyses across multiple sites and teams may be challenging due to the competing demands team members often face when their time is managed by multiple institutions (Taylor et al., 2018). This has certainly been the case for ICAN with most researchers based in study countries also working on other studies or tasks (e.g., teaching, administration) assigned by their respective institutions. Since the complexity of the ICAN study itself demands that many team members work on multiple project objectives, researchers have found themselves constantly trying to balance competing demands. As such, aspects of the project that are less urgent than others have sometimes slowed down or been relegated to just a few researchers due to team members' needs to prioritize and accomplish many tasks at once. For example, when reviewing and analyzing qualitative data for the Phase 2 rapid analysis only a few researchers conducted analysis of women's contraceptive decision-making, because other researchers were not available. While we were able to conduct this analysis relatively rapidly, the need to conduct rapid analysis meant that some researchers were excluded due to their limited availability. If we had been required to include all team members this would have significantly slowed the process.

Despite the multiple challenges that make rapid analysis generally more challenging in complex transnational studies and may undermine researchers' ability to conduct analysis that is truly “rapid,” we recommend that practitioners working to develop a new global health solution pair rigorous qualitative research with an HCD process. To do so, including rapid analysis of qualitative research in the process is necessary to ensure the iterative development of solutions is continually informed by data. In our project, we found that conducting in-depth qualitative research complemented the HCD process by adding rigor, and that the HCD process complemented our qualitative research by adding a focus on leveraging research to drive iterative solution development.

For studies that are able to integrate traditional qualitative analysis with rapid approaches to inform program design, we offer the following recommendations.

1. **Traditional qualitative and HCD researchers should work closely together from the beginning of the study to design qualitative tools that serve multiple purposes**. In our case, we used the PIRFs for both the traditional qualitative analysis, as well as our analysis to inform intervention design. These forms helped us to efficiently collect data that could immediately be used for multiple components of our study. In addition, HCD-related questions that were included in the qualitative interview guides also allowed us to gather more extensive data for this piece of the study than is normally used for HCD research, thus giving us a more substantial body of evidence on which to design programs.
2. **Carefully consider implementation logistics before developing data collection tools**. While the PIRFs were designed to be short and relatively easy to complete, for example, the memo templates ultimately went unused because they were too long and prescriptive.
3. **Establish accessible, shared spaces to store materials**. Our use of a shared Box drive made data transfer significantly easier and gave all team members both immediate and ongoing access to any materials produced in the analysis process that might inform other areas of analysis. Using the web-based platform Dedoose to code our data also facilitated data sharing and version control, and increased efficiency. Programs such as NVivo and Atlas.ti that require saving each coder's work individually and merging individual files would have required additional central management and made it more difficult for other team members to access the coded data.
4. **Consider the appropriate sequencing and combination of different types of data collection and analysis to accomplish multiple objectives efficiently and well**. In the ICAN study, we began with traditional formative qualitative data collection integrated with tools to facilitate rapid analysis. This gave us a set of full transcripts as well as notes to work with, both of which we were able to use for both a traditional qualitative analysis, as well as rapid analysis to inform program design.

In sum, our experience indicates that multiple factors such as linguistic and contextual differences may undermine researchers' ability to conduct analysis that is truly rapid. As global health studies become increasingly complicated and study teams often are pressed for both time and resources, some aspects of our approach may be particularly useful. Integrating traditional qualitative approaches with rapid analysis can be a highly efficient and effective way to meet multiple objectives of a complex study as long as the approach is carefully considered at the outset of the study and all team members have equitable opportunities to participate.

## ICAN Research Consortium

Kelsey Holt, Jenny X. Liu, Elizabeth Bukusi, Serah Gitome, Elizabeth Omoluabi, Address Malata, Peter Waiswa, Zachary Kwena, Louisa Ndunyu, Pauline Wekesa, Sarah Okumu, Ivan Idiodi, Chioma Okoli, Aminat Tijani, Ayobambo Jegede, Grace Nmadu, Shakede Dimowo, Martha Kamanga, Mandayachepa Nyando, Innocencia Mtalimanja, Tamandani Jumbe, Lynn Atuyambe, Dinah Amongin, Phoebe Alitubeera, Catherine Birabwa, Agnes Kayego, Betty Kaudha, Beth Phillips, Lauren Suchman, Janelli Vallin, Sneha Challa, Julia Kramer, Erica Sedlander, LaKia Williams.

## Data availability statement

The original contributions presented in the study are included in the article/Supplementary material, further inquiries can be directed to the corresponding author.

## Ethics statement

Ethical review and approval was not required for the study on human participants in accordance with the local legislation and institutional requirements. Written informed consent from the patients/participants or patients/participants legal guardian/next of kin was not required to participate in this study in accordance with the national legislation and the institutional requirements.

## Author contributions

LS conceived and designed the analysis, performed the analysis, and wrote the paper. EO, JK, JV, ES, SG, PW, and ZK contributed data or analysis, reviewed, and commented on drafts. RG, AK, BK, LA, DA, and PA contributed to analysis. AT, CO, AJ, MK, MN, LN, and KH contributed to analysis, reviewed, and commented on drafts. The ICAN Research Consortium contributed to analysis. All authors contributed to the article and approved the submitted version.
