# Supplementary material for: Analyzing fast and slow: Combining traditional and rapid qualitative analysis to meet multiple objectives of a complex transnational study
Source: Front Sociol. 2023 Feb 1;8:961202. doi: 10.3389/fsoc.2023.961202 (PMC9931144; doi:10.3389/fsoc.2023.961202)
Supplement: Supplementary file 1 [file Data_Sheet_1.docx]

**FOR INTERNAL USE**

**Purpose of IDIs:**

1. Deeply understand contraceptive decision-making & women’s experiences seeking, accessing, and using contraception, to understand for whom self-injection may be a powerful method
2. Ensure our working definition of “contraceptive autonomy” is salient to women, and understand how women themselves discuss related issues, as a precursor to item generation for the new contraceptive autonomy measure(s) to be validated in Nigeria and Uganda
   1. Working definition of contraceptive autonomy ([full definition here](https://docs.google.com/document/d/1HTpOmSLam9oMsiWoXQiVi3hzlrGi9YAJ/edit)):
      1. Consciousness of contraceptive rights and preferences (power within)
      2. Achievement of contraceptive rights and preferences (power to)
3. Identify women most interested in self-injection (SI), and their preferences for SI support (general and channel-specific), to guide the selection of user groups to focus on during ICAN and others’ program development work
4. Assess how COVID-19 has influenced women’s contraceptive decision-making and use, and women’s interest in SI as a contraceptive option

**Research Questions to be addressed through IDIs:**

1. **Domain 1- Contraceptive Decision-Making:**
   1. How do women form consciousness about contraceptive rights?
   2. How do women form contraceptive preferences?
   3. For whom might SI be a powerful method (even if they do not currently express interest)?
   4. How does women's consciousness of rights and preferences evolve over time? How do experiences acting on rights/preferences inform subsequent preferences and care-seeking? [Nigeria Cohort]
   5. How has the COVID-19 pandemic influenced women’s thoughts about and experiences with pregnancy and contraception?
2. **Domain 2- Contraceptive Use:**
   1. What influences whether and how women can act on contraceptive rights and preferences?
   2. For whom might SI be a powerful method (even if they do not currently express interest)?
   3. How does women's consciousness of rights and preferences evolve over time? How do experiences acting on rights/preferences inform subsequent preferences and care-seeking? [Nigeria Cohort]
   4. How has the COVID-19 pandemic influenced women’s thoughts about and experiences with pregnancy and contraception?
3. **Domain 3- Self-injection:**
   1. What are women's motivations and barriers for choosing SI, and what are the characteristics of women most interested in SI?
   2. Among SI users, what has their experience been with the method?
   3. What are women's preferences for SI provision/support?
   4. How do women's perspectives on SI change over time as the service provision landscape shifts? [Nigeria Cohort]
4. **Domain 4- Country-specific:**
   1. **KENYA:** What are women’s experiences and preferences related to purchasing self-care products online?
   2. **NIGERIA:**
      1. How do women navigate service-delivery options for contraception?
      2. How does women's consciousness of rights and preferences evolve over time? How do experiences acting on rights/preferences inform subsequent preferences and care-seeking? [Cohort]
   3. **UGANDA:**
      1. What are women’s experiences and preferences related to receiving contraceptive information and support from other women in a group setting?
      2. How does social communication with peers play a role in women’s ability to achieve contraceptive autonomy?
   4. **MALAWI:** What are women’s experiences and preferences related to interacting with health surveillance assistants (HSAs) for contraceptive care?

Study Title: Innovations for Choice and Autonomy (ICAN)

IRB No: SERU 4013

Version: 4.1 DATED 03 FEB 21

Session ID: DMPA_[_____]_[___ ___ ]_[__]_[_____] Date (DD/MM/YY): ____ /____ / ____

Name of Interviewer: ___________________________________________________________

**Before the Interview**

1. ***Introduce yourself and the study***
2. ***Make sure the participant is eligible***
3. Is the participant a woman? 🞏 YES 🞏 NO
4. Is she between 15-45 years of age? 🞏 YES 🞏 NO
5. Is she sexually active? 🞏 YES 🞏 NO
6. ***Obtain informed consent [READ CONSENT SCRIPT]***
7. Did you answer any questions she had? 🞏 YES 🞏 NO 🞏 N/A
8. Did she consent or assent to participate? 🞏 YES 🞏 NO🡪*STOP INTERVIEW*
9. *Optional:* Did she accept a copy of the consent form? 🞏 YES 🞏 NO
10. ***Obtain permission to audio record***
11. Did she give you permission to be recorded?

🞏 YES🡪*TURN ON RECORDER*

🞏 NO🡪*THANK HER AND CONCLUDE SESSION*

1. ***Turn on the tape recorder(s) and say:***

***“****Thank you for giving me permission to record, this is interviewer XXX and Session ID* XXX”

# Domain 1: Fertility and fAMILY PLANNING decision-making

## Part A: Fertility decision-making

[THE PURPOSE OF THIS SECTION IS TO UNDERSTAND HOW PEOPLE FORM INTENTIONS RELATED TO CHILDBEARING]

1. To start, can you please tell me a little bit about yourself?

🡺 ***[PROBE]:***  *Tell me about yourself in regard to when you were born, what number you are in your family, about your siblings, parents, your education level, what you do etc]*

- 1. Who do you live with?

1. Can you describe your role in your family/household?

Now, I’d like to talk about how you make small and large decisions about your life.

1. Think back to the last time you made a small decision that affected you. What was the decision about and how did it get made? For example, a decision about whether or not to go visit a friend of yours who lives nearby.

- 1. Please walk me through how the decision was made
  2. Who else was involved in the decision?
  3. Why was [person/people] involved in the decision?
  4. What did you agree on?
  5. What did you disagree on?
  6. How did you resolve any disagreement?
  7. What opposition did you face?

🡺 ***[PROBE]:*** What did you do?

- 1. How did you feel about your role in making the decision?

🡺 ***[PROBE]:*** What role do you wish you had played instead?

1. Think back to the last time you made a big decision that affected you. What was the decision about and how did it get made? For example, a decision related to spending a large sum of money OR whether to travel outside of your community.

- 1. Please walk me through how the decision was made
  2. Who else was involved in the decision?
  3. Why was [person/people] involved in the decision?
  4. What did you agree on?
  5. What did you disagree on?
  6. How did you resolve any disagreement?
  7. What opposition did you face?
  8. How did you feel about your role in making the decision?

🡺 ***[PROBE]:*** What role do you wish you had played instead?

1. You mentioned that you have [X] children. Please tell me what reasons you have for having (x) number of children.

🡺 ***[PROBE]:*** *What other reasons are there for having (x) number of children?*

1. Do you feel you personally **have had the freedom** to decide whether to have children at all?
   1. What makes you say that?
   2. **What would happen if you didn’t want to have children**?
2. Do you feel you personally **have had the freedom** to decide when to have children?
   1. What makes you say that?
3. How do you feel about how much freedom you have to decide whether or when to have children?
4. When it comes to deciding whether or when to have children, do you think there is one person in a family whose opinion is most likely to determine what the family decides to do?
   - 1. Why do you think this?
5. Do you want [more] children someday?

🡺 ***[PROBE]:* (If the participant is pregnant)** Would you like to have more children after your current pregnancy?

- - 1. **IF YES OR UNSURE:**
       - 1. Why do you feel that way?
         2. How many more children might you like to have?
         3. When would you like to have more children?
    2. **IF NO:** Why do you feel that way?

1. Thinking back over your lifetime, who are the most important people you have talked to about if you would have children?

🡺 ***[PROBE]:*** Anyone else?

🡺 ***[PROBE]:*** *When have you had these conversations?*

- - 1. Why do you feel these are the most important people to talk to about whether or not to have children?
    2. ***If you haven’t talked to anyone about childbearing,*** why is that so?

1. Please think back to one of the most important conversations you’ve had about if you would have children. Can you tell me about how that conversation went?
2. Who have you avoided talking to about if you would have children?

🡺 ***[PROBE]:*** *Anyone else?*

- - 1. Why have you avoided talking to [this person / these people] about if you would have children?

## Part B: Family planning decision-making

[THE PURPOSE OF THIS SECTION IS TO UNDERSTAND HOW PEOPLE FORM INTENTIONS RELATED TO USING (OR NOT USING) FAMILY PLANNING]

1. If a woman/girl wanted to prevent pregnancy, what could she do? [THIS QUESTION IS TO GIVE BRIEF CONTEXT TO WHAT SHE VIEWS AS PREGNANCY PREVENTION]

🡺 ***[PROBE]:*** *Anything else?*

1. Where have you learned about these methods of pregnancy prevention?

**🡺 *[PROBE]:*** *What have you learned about pregnancy prevention from:*

- *Friends?*
- *Family?*
- *Other people?*
- *Things you have heard on TV, radio, or internet?*
- *Churches/mosques*
- *Schools/community forum*

1. Tell me about the rights women should have related to pregnancy prevention. By “rights” I mean the things women *should* have access to and *should* be able to do related to pregnancy prevention
   1. If a woman **wants to access information** about pregnancy prevention, are there any circumstances in which she shouldn’t be allowed to?

▪  Why do you think that?

- 1. **If a woman wants** to make a decision by herself about whether to use a pregnancy prevention method, should she be allowed to?

▪  Why do you think that?

What is or has been your own experience with making decisions about contraception for yourself?

- 1. Do you feel entitled to make these decisions?
  2. If you want to **make a decision about contraception for yourself**, do you feel that other people in your life have to **allow you to do so**?
  3. Why do you feel that way?

1. Have you ever considered doing something to prevent pregnancy?
   - 1. **IF YES:**
     2. At what points in your life did you think about preventing pregnancy?
     3. Tell me about one of those times – When and why did you think about preventing pregnancy?
     4. Who did you talk to about it?

**🡺 *[PROBE]:*** What did you think people in your life would think of you if you started using contraception?

- - 1. What did you end up doing?
    2. Why did you take that approach?
    3. How did you feel about what you ended up doing?
    4. **IF NO:**
    5. Why have you not done anything to prevent pregnancy?

**🡺 *[PROBE]:*** What would the people in your life think about you if you started doing something to prevent pregnancy?

- - 1. What else has influenced your thinking about not doing anything to prevent pregnancy?
    2. How has it influenced you?

1. Apart from you, who else has a say in whether or not you do something to prevent pregnancy?

**🡺 *[PROBE]****: Anyone else?*

**🡺 *[PROBE]****: If no one has a say, why do you think so?*

- - 1. Please tell me more about how [this person/these people] has influence.

**🡺 *[PROBE]:*** *Can you give me an example of a conversation you’ve had with [this person/these people] about preventing pregnancy?*

- - 1. How do you feel about the fact that [this person/these people] has influence?
    2. What might happen if you didn’t follow their advice?
    3. Do you have to get anyone’s approval to do something to prevent pregnancy?

**🡺 *[PROBE]:*** *Please explain*

**🡺 *[PROBE]:*** *How do you feel about this?*

**IF NO:** *Why?*

- - 1. Has anyone ever tried to stop you from doing something to prevent pregnancy?

**🡺 *[PROBE]:*** *If yes: Please explain*

**🡺 *[PROBE]:*** *If yes: How did you feel about this?*

- - 1. Has anyone ever tried to pressure you into doing something to prevent pregnancy?

**🡺 *[PROBE]:*** *If yes: Please explain*

**🡺 *[PROBE]:*** *If yes: How did you feel about this?*

1. What should a woman do if her husband does not agree to her family planning choice?
   1. What do you think about a person using contraception in secret from their partner?
   2. What makes you think this way?
2. Who is the most important person that you want to be involved when you decide whether or not to do something to prevent pregnancy?
   - 1. Why do you want this person to be involved (or why do you want no one involved)?

*If the participant wants someone to be involved:*

- - - 1. How do you want them to be involved?
      2. Would they want to be involved?

**🡺 *[PROBE]:*** Why or why not?

1. Is there anyone in your life who you would NOT want to know that you are doing (or have done) something to prevent pregnancy?
   - 1. **IF YES:**
        - 1. Who?
          2. How is your relationship with that person?
          3. Why would you NOT want this person to know that you are doing (or have done) something to prevent pregnancy?
          4. How does that make you feel?
          5. Is there any way that they could find out?
          6. What would happen if they found out?
     2. **IF NO:**
        - 1. Why is that?
2. Have you heard of the recent COVID-19 pandemic?
   - 1. **IF YES: You mentioned that you are doing/ not doing something to prevent pregnancy**
        - 1. How has the COVID pandemic affected your thoughts about whether or not you’d like to do something to prevent pregnancy right now?
          2. The COVID-19 pandemic has affected many people in different ways. How has the COVID pandemic affected your thoughts about pregnancy?
     2. **IF NO:**
        - 1. The COVID-19 pandemic is a contagious virus that is spreading, which has led to restrictions on travel and gatherings of large groups of people. Have you been affected by this?

**IF YES:**

How has it affected your thoughts about pregnancy?

How has it affected your thoughts about whether or not you’d like to do something to prevent pregnancy right now?

**IF NO:** [CONTINUE WITH DOMAIN 2]

# Domain 2: family planning Use

[THE PURPOSE OF THIS SECTION IS TO UNDERSTAND BARRIERS AND FACILITATORS TO WOMEN/GIRLS ACTING ON THEIR INTENTIONS TO USE OR NOT USE FAMILY PLANNING]

1. You have told me a little about your experience with pregnancy prevention already. Now, I want to be sure I have a full picture of the family planning methods you’ve ever used.
   - 1. What method, if any, are you using now?
     2. Which of these other methods **[SHOW MODELS]**, if any, have you used in the past? [BE SURE TO VERBALIZE FOR RECORDING EACH THEY INDICATE HAVING USED]
     3. Sometimes people use other techniques to prevent pregnancy that don’t require using a device or taking a medicine. These include things like pulling out before a man ejaculates, timing when you have sex to avoid the most fertile times, or relying on breastfeeding to keep you from getting pregnant. People sometimes also take traditional herbs because they believe these prevent pregnancy. Is there anything else like this you’ve ever used to prevent pregnancy?
     4. **IF USED INJECTABLE Family planning:** Have you ever injected yourself?

[IF YES, NOTE THAT A SERIES OF QUESTIONS IN DOMAIN 3 WILL PROBE ABOUT HER EXPERIENCE]

INTERVIEWEE **HAS** USED A METHOD TO PREVENT PREGNANCY

| ***CONTINUE TO A*** |
| --- |

INTERVIEWEE **HAS NEVER** USED A METHOD TO PREVENT PREGNANCY

| ***SKIP TO B*** |
| --- |

## INTERVIEWEE HAS USED Family planning:

Now, I’d like to talk about your experience with pregnancy prevention. I’m going to ask you a series of questions about your experience using and accessing family planning.

**IF HAS USED INJECTABLE FAMILY PLANNING BEFORE:** You mentioned that you have used injectable family planning before, I’d like to hear about that.

**IF HAS NOT USED INECTABLE FAMILY PLANNING BEFORE:** Please select the method that you feel you have the most to say about to answer the questions. Remember this can include so-called “modern” methods that require a device or medicine, but also things like timing when you have sex to avoid your fertile period.

1. Thinking about this method, could you tell me about your experience using it?

**🡺 *[PROBE]:*** *How long did you use it?*

**🡺 *[PROBE]:*** *What do you like about this method? Why?*

**🡺 *[PROBE]:*** *What don’t you like about this method? Why?*

1. Before you started using this method, where did you hear about it?
   - 1. What information did you have about it before you started using it?
2. Please tell me your reasons for starting to use this particular method instead of any other.
   1. Who did you talk to about this particular method?
   2. How did they influence your decision?
   3. Did you have to get anyone’s approval to use this method?

**🡺 *[PROBE]:*** *If yes, did you get their approval? How did that happen?*

**🡺 *[PROBE]:*** *If no, why not?*

- 1. Did anyone try to influence you to use this method?

**🡺 *[PROBE]:*** *If yes, please explain*

- 1. When choosing this method, was HIV or any other sexually transmitted infection ever a concern? How did your concerns about HIV and other sexually transmitted infections influence your decision on choosing this family planning method?

**🡺 *[PROBE]:*** *If yes, what was the concern? How did that influence your decision?*

**🡺 *[PROBE]:*** *If no, why not?*

1. Tell me about the first time you obtained this method.
   - 1. How did you decide where to go?

**🡺 *[PROBE]:*** *What else influenced where you went?*

- 1. What was difficult about getting it?
  2. What was easy about getting it?
  3. How expensive was it?
  4. How did you get enough money for it?
  5. How did the person who gave it to you treat you?
  6. What kinds of questions did the person ask you?
  7. Did the person who gave it to you tell you about other options for preventing pregnancy?
     - 1. **IF YES:** What did they tell you?
  8. What did the person who gave it to you tell you about how to use the method?

1. Since that first time, tell me about other experiences you had with refilling the method or having follow-up appointments?
   1. What has been good about those experiences?
   2. What has been bad about those experiences?
   3. How did the price change?
   4. Did it get more or less convenient? Why?
   5. How has the COVID-19 pandemic influenced your ability to get a refill or follow-up support for this method?
2. How much power or control of your life [did you/do you] feel when using this method?
   - 1. Why do you feel that way?
     2. What would make you feel like you are in more control?
3. Did you ever take a break from using this method and then come back to it?

**🡺 *[PROBE]:*** Why did you decide to take a break?

1. **IF STILL USING THIS METHOD:** Why are you continuing to use this method instead of something else?
   - 1. How confident do you feel that you could continue to use this method as long as you wanted to?

**🡺 *[PROBE]:*** *Why do you feel that way?*

1. **IF NO LONGER USING THIS METHOD:** Why did you stop using this method?
   1. What other reasons did you have for stopping the method?
   2. Who did you talk to about stopping using the method?
   3. How did they influence your decision?
   4. How did that make you feel?
2. **IF HAS EXPERIENCE WITH MORE THAN ONE METHOD:** Is there anything else you want to tell me about how your experience with [the method we’ve been talking about] compares with your experience using and accessing other contraceptive methods?

**🡺 *[PROBE]:*** What was easier about using this method compared to other methods?

**🡺 *[PROBE]:*** What was harder about using this method compared to other methods?

1. **IF NOT CURRENTLY USING Family Planning:** Under what circumstances might you start using contraception again in the future?
   - 1. If you wanted to start using Family Planning again, how confident do you feel that you could?

**🡺 *[PROBE]:*** *Why do you feel that way?*

- - 1. What method do you think you would use if you started using Family Planning again?
  1. Why would you choose that method?
  2. Where would you go to get it?
  3. Why would you get it from there?

| ***SKIP TO DOMAIN 3*** |
| --- |

## INTERVIEWEE HAS NEVER USED Family Planning:

1. If you decided you wanted to use something to prevent pregnancy, how confident are you that you could do so?
   - 1. Why do you feel that way?
     2. What else would affect your ability to do something to prevent pregnancy if you wanted to?
2. If you decided you wanted to use something to prevent pregnancy, what method do you think you might use?
   - 1. Why this particular method instead of another one?
     2. Where would you go to obtain the method?
     3. How easy or difficult do you think it would be to get that method?

# Domain 3: Self-injection

[THIS SECTION IS MEANT TO UNDERSTAND WOMEN’S INTEREST IN SELF-INJECTION AND, FOR THOSE WHO HAVE EXPERIENCE, HOW IT HAS BEEN]

1. Are you aware of any medical/health products that you can inject by yourself at home (without the help of a medical provider or pharmacist)?
   1. **IF YES:**
      1. Which products are these?
      2. What do you know about these products?
      3. How did you hear about them?
      4. Are there any other products you are aware of?
      5. Have you ever used any of these products?
      6. Could you describe what this product looked like?

**PROCEED IF INTERVIEWEE HAS SELF-INJECTED DMPA-SC;
OTHERWISE SKIP TO QUESTION 53**

*Now I am going to ask you questions about Sayana Press. Sayana Press is an injectable family planning method that someone can buy from a pharmacy or get from a health facility and inject themselves every three months. It looks like this* ***[SHOW THE PARTICIPANT THE SAYANA PRESS MODEL]***

1. How many times have you injected yourself with Sayana Press?
2. When you received Sayana Press, what were you told about the possibility of injecting yourself?
   1. Were you told you could do the injection yourself?
   2. Were you offered the option of taking home doses to inject yourself with after your initial shot?
   3. What else were you told about the possibility of self-injection?
3. How has it been injecting yourself with Sayana Press?
4. What is easy about it?
5. What is difficult about it?
6. How has disposing of the needle been for you?
7. Were you taught how to inject yourself?
8. **IF YES**:
9. Who trained you?
10. Can you tell me how [trainer] taught you how to inject yourself?
11. What did the [trainer] say about how to know when to take your next dose?
12. What did the person say about disposing the needle?
13. What did you like about the training?
14. What didn’t you like about the training?
15. What additional information or support would have been helpful?
16. **IF NO**:
17. Why did you not receive training?
18. What additional information or support would have been helpful?

**IF PARTICIPANT IS USING SAYANA PRESS (DMPA-SC) BUT NOT SELF-INJECTING, GO TO QUESTION 43**

**INTERVIEWEE IS USING SAYANA PRESS AND IS SELF-INJECTING**

1. How does injecting yourself make you feel?
2. Why does it make you feel that way?
3. What does injecting yourself enable you to do that you couldn’t otherwise do if you had to go see a health provider each time?
4. Who in your life knows that you are self-injecting?
   - 1. Who else?
     2. Is there anyone you have kept it secret from?

**🡺 *[PROBE]: Why is that?***

- - 1. How have others in your life reacted to the fact that you are injecting yourself?
    2. How does this make you feel?

1. How confident do you feel that you could continue to inject yourself with Sayana Press if you wanted to?
   - 1. Why?
2. Do you plan to continue injecting yourself with Sayana Press?
   - 1. Why/why not?

**🡺 *[PROBE]:*** How does self-injection fit or not fit with your life right now?

- 1. **IF YES, PLANS TO CONTINUE SELF-INJECTING:**
     - 1. Where do you plan to get the refills next time?
       2. Why that person/place?
       3. What other help would you like in the future with injecting yourself?
       4. How many 3-month doses would you want to take home at a time?

**SKIP TO QUESTION 49**

**INTERVIEWEE IS USING SAYANA PRESS BUT NOT SELF-INJECTING**

1. How many times have you been injected with Sayana Press?
   1. Who has been injecting you?
   2. Is this every time you received a shot of Sayana?
   3. Why this person(s)?
   4. Would you consider being injected by another woman in your community that uses Sayana Press?
2. What has been your experience with being injected with Sayana Press?
   1. What has been easy?
   2. What has been hard?
3. Why have you not tried injecting yourself with Sayana Press?

a. Why do you feel that way?

b. What other concerns might you have about injecting yourself?

c. What help would you like to help you practice self-injection with Sayana Press?

i. Is there anything else?

1. Who in your life knows that you are using Sayana Press?
   1. Who else?
   2. Is there anyone you have kept it secret from?

**🡺 *[PROBE]:*** *Why is that?*

- 1. How have others in your life reacted to the fact that you are using Sayana Press, (*but not self-injecting*)?

**🡺 *[PROBE]:*** *Colleagues, friends, family, health worker*

- 1. How does this make you feel?

1. How confident do you feel that you will ever inject yourself with Sayana Press?
   1. Why or why not?
2. Do you plan to continue using Sayana Press?
   1. Why or why not?

**FOR ALL INTERVIEWEES USING SAYANA PRESS**

1. What are the benefits you see to self-injecting contraception rather than going to a health care provider or pharmacist for an injection?
   - 1. Any other benefits?
2. What are the challenges of self-injecting rather than going to a health care provider or pharmacist for an injection?
   - 1. Any other challenges?
3. Who do you think might most benefit from self-injectable contraception?
   - 1. Why this person/these people?
4. Would you recommend Sayana Press to other women?
   - 1. Why/why not?
     2. Do you think people you know would be interested in injecting themselves with Sayana Press?
        - 1. Why/why not?

**SKIP TO DOMAIN 4**

**IF THE INTERVIEWEE HAS NOT HEARD ABOUT SAYANA PRESS**

1. Scientists have developed a device that people can use at home to inject themselves with medicines like those that are used to treat diabetes **[SHOW UNIJECT DEVICE, DESCRIBE SELF-INJECTION].**  How would you feel about keeping medicines at home and injecting yourself with them when you needed them?
2. One use of these devices is a contraception for pregnancy prevention. This is called “Sayana Press” and a person can inject themselves every three months to prevent pregnancy. If you needed contraception, would you be interested in injecting yourself with Sayana Press?
   - 1. Why/why not?

**🡺 *[PROBE]:*** Do you think people you know would be interested in injecting themselves with Sayana Press?

- - - - 1. Why/why not?
    1. **FOR CURRENT USERS OF ANY CONTRACEPTIVE METHOD:**
       - 1. Would you be interested in using Sayana Press instead of your current method?
         2. Why/why not?

**IF YES:**

- - - - 1. How much would you be willing to pay for Sayana Press?
        2. Where would you like to obtain Sayana Press?

1. If you wanted to inject yourself with Sayana Press, how would you like to learn how to inject yourself?

**🡺 *[PROBE]:*** *For example, going to the healthcare clinic, a pamphlet, online video/post*

1. Why would you like to learn this way?
2. Who would you like to be involved in helping you learn?
3. What are the questions you would want answered regarding self-injection?
4. How many 3-month doses would you want to take home at a time?
5. What are the benefits you see to self-injecting contraception rather than going to a health care provider or pharmacist for an injection?
   - 1. Any other benefits?
6. What are the challenges of self-injecting rather than going to a health care provider or pharmacist for an injection?
   - 1. Any other challenges?
7. Who do you think might most benefit from self-injectable contraception?
   - 1. Why this person/these people?

# Domain 4: e-commerce platforms

[THIS SECTION IS MEANT TO EXPLORE HOW E-COMMERCE CAN BEST MEET WOMEN’S CONTRACEPTIVE NEEDS]

1. Have you ever used a website or app on your phone to do any online shopping?
   1. **IF NO:**
      1. Why have you not purchased anything on a website or app on your phone?

**🡺 *[PROBE]:*** What are the barriers you face to shopping online?

- - 1. If you wanted to order something online, how easily could you find a computer or phone to use?

- - 1. If you wanted to order something online, how confident are you that you would be able to use the website or app?
  1. **IF YES:**
     1. What have you bought online?

Have you ever used a website or app to order personal care products, such as perfumes or cosmetics or feminine hygiene products such as pads or tampons?

- - 1. Have you ever used a website or app to order contraception, including condoms, pills, or other methods?
    2. What app(s) have you used?
       - 1. Why did you choose this/these app(s)?
    3. If you have used a website or app to order a personal care product or contraceptive method, please answer these next questions about that. Otherwise, feel free to answer about your other online ordering experiences.
       - 1. Why did you buy these products online rather than go to a store or clinic?

**🡺 *[PROBE]:*** What are the advantages of online vs. in-person shopping?

**🡺 *[PROBE]:*** What was easier about this than going to a store or clinic?

- - - - 1. What was harder about this experience compared to going to a store or clinic for the product(s)?
        2. What did you like about the website or app?
        3. What did you dislike about the website or app?
        4. How frequently do you use it for such purchases?
        5. What was the payment experience like?
        6. How could the experience have been improved for you?

1. **IF HASN’T ALREADY REPORTED PURCHASING PERSONAL CARE PRODUCTS ONLINE:** Are you aware of any websites or apps that allow you to order personal care products or contraception online?
   - 1. **If yes:** Is there a reason why you haven’t used these kinds of websites/apps before?
     2. What would make you want to use this kind of website/app for ordering contraception?
     3. Would you feel comfortable using a website or app to order contraception?
        - 1. What makes you feel that way?
2. Have restrictions around COVID-19 affected the way you shop for personal care products at all?
   - 1. **If yes:** How?
3. Do you have any other comments to add as we conclude the interview?

*Thank you for participating in this study. We appreciate your time.*

**TURN OFF RECORDER**
