# Supplementary material for: Analyzing fast and slow: Combining traditional and rapid qualitative analysis to meet multiple objectives of a complex transnational study
Source: Front Sociol. 2023 Feb 1;8:961202. doi: 10.3389/fsoc.2023.961202 (PMC9931144; doi:10.3389/fsoc.2023.961202)
Supplement: Supplementary file 2 [file Data_Sheet_2.doc]

ICAN Study Post-interview Report Form

|  | | |
| --- | --- | --- |
| **Interview information** | | |
| **Interview date & time** |  | |
| **Location** |  | |
| **Participant ID #** |  | |
| **Interviewer** |  | |
|  | | |
|  | | |
| **Interview guide** | | |
| 1. **How did the interviewee describe their contraceptive decisions?  (Consider when the interviewee started using contraception, how they decided to use contraception, and how they settled on their current contraceptive method.)** | |  |
| 1. **How did the interviewee describe the process of procuring contraception?  (Consider where the interviewee currently accesses their contraception and if they are satisfied with their current way of accessing contraception.)** | |  |
| 1. **What things did the interviewee mention that made it easier to achieve their contraceptive preferences/choices?** | |  |
| 1. **What things did the interviewee mention that made it harder to achieve their contraceptive preferences/choices?** | |  |
| 1. **What did the interviewee think about self-injection?** | |  |
| 1. **Was there anything surprising about the interview?** | |  |
| 1. **Did you face any problems or challenges in the interview?** | |  |
| 1. **Are there any changes we should consider making to the interview guide? (examples: changing the wording of questions, adding NEW questions or follow-up questions, changing the order of questions)** | |  |
