# Supplementary material for: Analyzing fast and slow: Combining traditional and rapid qualitative analysis to meet multiple objectives of a complex transnational study
Source: Front Sociol. 2023 Feb 1;8:961202. doi: 10.3389/fsoc.2023.961202 (PMC9931144; doi:10.3389/fsoc.2023.961202)
Supplement: Supplementary file 3 [file Data_Sheet_3.docx]

**Weekly Team Meeting Template**

|  | | |  |
| --- | --- | --- | --- |
| **Meeting information** | | |  |
| **Meeting date & time** |  | |  |
| **Attendees** |  | |  |
|  | | |  |
|  | | |  |
| **Content Common Themes** | | | **Surprising Findings** |
| 1. **How did interviewees describe their contraceptive decisions?  (Consider when interviewees started using contraception, how they decided to use contraception, and how they settled on their current contraceptive method.)** | |  |  |
| 1. **How did the interviewees describe the process of procuring contraception?  (Consider where interviewees currently accesses their contraception and if they are satisfied with their current way of accessing contraception.)** | |  |  |
| 1. **What things did the interviewees mention that** **made it easier** **to achieve their contraceptive preferences/choices?** | |  |  |
| 1. **What things did the interviewees mention that** **made it harder** **to achieve their contraceptive preferences/choices?** | |  |  |
| 1. **What did the interviewees think about self-injection?** | |  |  |

| **Process** | |
| --- | --- |
| 1. **Were there any problems or challenges that came up in multiple interviews?** |  |
| 1. **Are there any changes we should consider making to the interview guide? (examples: changing the wording of questions, adding NEW questions or follow-up questions, changing the order of questions)** |  |
| 1. **Should we consider talking to any NEW groups of people? If so, who and why?** |  |
| 1. **Are there any issues from any of the interviews that need to be brought to the attention of the research team?** |  |
